# Screening patients for unintentional carbon monoxide exposure in the Emergency Department: a cross-sectional multi-centre study

**DOI:** 10.1093/pubmed/fdad007

**Published:** 2023-01-31

**Authors:** Heather Jarman, Richard W Atkinson, Desislava Baramova, Timothy W Gant, Tim Marczylo, Isabella Myers, Shirley Price, Tom Quinn

**Affiliations:** Emergency Department Clinical Research Group, St George’s University Hospitals NHS Foundation Trust, London SW17 0QT, UK; Population Health Research Institute, St George’s, University of London, London SW17 0RE, UK; Centre for Health and Social Care Research, Kingston University, Kingston KT1 1LQ, UK; Population Health Research Institute, St George’s, University of London, London SW17 0RE, UK; Emergency Department Clinical Research Group, St George’s University Hospitals NHS Foundation Trust, London SW17 0QT, UK; Radiation, Chemical and Environmental Hazards, UK Health Security Agency, Oxford OX11 0RQ, UK; Radiation, Chemical and Environmental Hazards, UK Health Security Agency, Oxford OX11 0RQ, UK; University of Surrey, Surrey GU2 7XH, UK; University of Surrey, Surrey GU2 7XH, UK; Centre for Health and Social Care Research, Kingston University, Kingston KT1 1LQ, UK

**Keywords:** Carbon monoxide, emergency, toxicology, poisoning, public health

## Abstract

**Background:**

Low-level exposure to carbon monoxide (CO) is a significant health concern but is difficult to diagnose. This main study aim was to establish the prevalence of low-level CO poisoning in Emergency Department (ED) patients.

**Methods:**

A prospective cross-sectional study of patients with symptoms of CO exposure was conducted in four UK EDs between December 2018 and March 2020. Data on symptoms, a CO screening tool and carboxyhaemoglobin were collected. An investigation of participants’ homes was undertaken to identify sources of CO exposure.

**Results:**

Based on an ED assessment of 4175 participants, the prevalence of suspected CO exposure was 0.62% (95% CI; 0.41–0.91%). CO testing in homes confirmed 1 case of CO presence and 21 probable cases. Normal levels of carboxyhaemoglobin were found in 19 cases of probable exposure and in the confirmed case.

**Conclusion:**

This study provides evidence that ED patients with symptoms suggestive of CO poisoning but no history of CO exposure are at risk from CO poisoning. The findings suggest components of the CO screening tool may be an indicator of CO exposure over and above elevated COHb. Clinicians should have a high index of suspicion for CO exposure so that this important diagnosis is not missed.

## Introduction

Carbon monoxide (CO) is a colourless, tasteless, odourless gas formed by incomplete combustion of carbon compounds. It is reported as one of the most common causes of death from poisoning worldwide.^1^ Unintentional non-fire related CO exposure was reported as a cause of death in 20 patients in England and Wales in 2020 and results in 4000 visits to Emergency Departments (ED) in the UK each year.^2^^,^^3^ Comparable rates are reported in other countries with higher incidence in lower socioeconomic groups.^4–7^ Common sources of CO found in indoor environments are incorrectly installed, poorly maintained, inappropriately used, or poorly ventilated fossil fuel and wood burning heating and cooking appliances.^8–10^ CO poisoning is diagnosed by a clinical triad: symptoms consistent with CO poisoning, history of recent CO exposure and elevated carboxyhaemoglobin (COHb) levels.^11^^,^^12^

Prevalence of occult unintentional CO poisoning in patients presenting to EDs ranges from 0.1 to 7.1%.^13^^,^^14^ Studies consistently identify under-recognition and therefore under-reporting of unintentional CO poisoning, making official figures a known underestimate of the true prevalence.^15–17^

We which aimed to link all three elements of the diagnostic triad of CO poisoning to ascertain the number of patients presenting to the ED with symptoms suggestive of unintentional non-fire-related CO poisoning linked to CO exposure from a domestic source.

### Study objectives

1. To determine the proportion of patients presenting to ED with symptoms suggestive of CO exposure that have raised COHb levels, and to investigate the proportion that could be linked to possible CO exposure from a domestic appliance source.
2. To validate a tool for screening for carbon monoxide exposure.^18^^,^^19^

## Methods

### Study design and setting

A cross-sectional observational study was conducted (Supplementary Material 1). Patients presenting to the EDs of four UK hospitals in the South of England with symptoms associated with low-level carbon monoxide exposure between December 2018 and March 2020 were recruited. The study was approved to take place in the UK NHS by the London Queen Square Research Ethics Committee (REC No: 18/LO/1381) in October 2018, trial registration number: ISRCTN16329899.

### Study participants

Adults aged 18 years or over who were admitted to the ED with symptoms suggestive of CO exposure (cardiac chest pain, non-traumatic headache, flu-like symptoms, seizures and syncope) were included (Supplementary Material 2). Patients who attended with smoke inhalation from fire-related incidents were excluded as the condition of interest was low-level unintentional CO exposure.

Patients were identified on arrival to the ED by clinical or research staff. Each participant had blood drawn for point of care analysis of COHb as soon as possible after arrival. COHb levels were obtained from a venous blood gas sample through a point of care test (POCT) analyser as part of routine by clinical staff. To expedite rapid measurement of carboxyhaemoglobin (COHb), patients gave verbal consent for blood samples to be taken with written consent sought subsequently.

Data were collected on a standardized reporting form. This included details of symptoms and self-reported smoking status. Participants were screened using the four-question ‘COMA’ screening tool as the recommended first step to guide the diagnosis of CO poisoning in primary and emergency care in the UK.^18^^,^^19^ The questions/screening criteria are as follows:

1. COMA_C: Cohabitees/companions: Is anyone else in the property affected including pets?
2. COMA_O: Outdoors: Do your symptoms improve when out of the building? (‘better outdoors’)?
3. COMA_M: Maintenance: Are your fuel burning appliances and vents properly maintained?
4. COMA_A: Alarm: Do you have a carbon monoxide alarm?

If the participant answered yes to both or either of the first two questions, or no to both or either of the last two questions, then the patients were classified as COMA positive and the clinician was prompted to consider CO exposure and take appropriate clinical measures. Patients that responded no to both of the first two questions and yes to both the last two were classed as COMA negative.

Patients were assessed and managed in line with local processes at each site. In accordance with UK Health and Safety Executive and clinical guidance for reporting of a potential CO exposure, participants with raised COHb or symptoms consistent with CO exposure (based on cohabitees being affected or symptoms resolving if outdoors) were advised to contact the National Gas Emergency Service (NGES) to assess for CO and potential sources of CO from faulty appliances in their home.^2^^,^^20^ For patients who were unable to contact NGES themselves, this was done for them by ED staff. Emergency gas safety engineers are required to investigate the scene and make appliances (irrespective of fuel type) safe within 2 hours of the notification and the referral was made as soon as possible after CO exposure was identified to ensure risk to others who may be affected in the property was reduced.

A slightly lower cut-off value, compared with the clinical definition for COHb levels (>2% in non-smokers, > 10% in smokers), was used for determining who should contact NGES, with the study steering group selecting > 1.6% for non-smokers and > 6.3% for smokers. This lower level used was to ensure no cases were missed.

Participants in the study consented for the routinely collected information during this inspection by certified gas safety engineers to be shared with the study team; this included environmental readings of CO and results of any checks on gas appliances.

### Variables

The variables required to assess CO exposure were as follows:

- Symptoms consistent with exposure, including screening questions on CO exposure risk using the ‘COMA’ tool.
- Raised COHb levels: COHb levels were obtained from a venous blood gas sample processed through a POCT blood gas analyser (ABL90 flex/plus Radiometer or ABL800 flex Radiometer) at each ED according to standard hospital procedures. All analysers were calibrated in line with manufacturer’s instructions and established quality control procedures within each site. Venous COHb was used as the biomarker of CO detection as it is routinely used in EDs in the UK and recommended in guidance on assessment of CO poisoning.^14^^,^^18^ The cut-off values for COHb used in the analysis were those above the normal upper limits recommended by the UK National Poisons Information Service database (TOXBASE) of 2% in non-smokers and 10% in smokers^20^
- Detection of confirmed or probable presence of CO in the home: NGES documentation of the presence of CO as a confirmed source or the presence of a faulty appliance that could be responsible for CO leak as a probable source were used. A probable source definition was used in this study as CO dilutes from the indoor environment through ventilation with ambient air and therefore may not be detected during monitoring or following ventilation of the property.

### Data sources/measurement

Participants were identified as having suspected, probable and confirmed exposure based on the case definitions in Table 1.

**Table 1:** Case definitions for CO exposure

Suspected	Probable	Confirmed
Symptoms suggestive of CO exposure AND COHb levels > 2% (non-smokers) and > 10% (smokers) and/or Self-report ‘Yes’ to C or O on the COMA screening tool	Symptoms suggestive of CO exposure AND COHb levels > 2% (non-smokers) and > 10% (smokers) and/or Self-report ‘Yes’ to C or O on the COMA screening tool AND Environmental report of the possible presence of CO (faulty or potentially faulty appliance)	Symptoms suggestive of CO exposure AND COHb levels > 2% (non-smokers) and > 10% (smokers) and/or Self-report ‘Yes’ to C or O on the COMA screening tool AND Environmental report of the presence of CO

#### Bias

Bias was minimized in the study by collecting data through standardized reporting and assessment of measures and using a recognized screening tool. All research staff collecting data were provided with study specific training on study procedures and data collection.

Self-reported smoking status was used, which may be subject to social desirability bias and false report of non-smoking in some participants.

#### Study size

A previous study estimated the prevalence of unexpectedly high COHb in an English ED population to be 4.3%.^16^ Based on the attendance rates of patients with the known conditions of interest observed in this study and a predicted recruitment rate of approximately 40%, an estimated sample of 5222 would be required to identify 235 participants with raised COHb. We anticipated recruitment rates across the four sites to be around 2500 per year over a 2-year period.

### Statistical methods

The proportion (exact 95% confidence interval) of probable cases in each exposure group was calculated. The small number of subjects with missing COHb measures or COMA responses was excluded from the analyses. Pearson Chi-square test was used for comparison of proportions between groups. Data analyses were conducted in STATA StataCorp. 2017. Stata Statistical Software: Release 15. College Station, TX: StataCorp LLC.

#### Patient and public involvement

Members of a patient and public research expert group were involved in the design of the study and provided advice on consent procedures and content of the patient information material. Representatives from gas safety organizations were on the steering committee.

## Results

### Participants

In total, the four sites identified 6915 participants who met the inclusion criteria, of which 4392 (63.5%) were enrolled into the study. Of the 2523 participants not enrolled, the most common reason (33%) was the participant having a non-cardiac cause of chest pain, and 686 (27.2%) were unwilling or unable to participate. Incorrect calibration of the blood gas analyser at one of the hospitals meant that 200 participants were removed as their COHb results could not be confirmed as accurate, a further 2 withdrew consent after blood sampling and COHb measurements for a further 15 participants were not recorded. Hence, a total of 4175 participants were included in the study. The planned target of 5222 was not achieved as the study ended recruitment early due to the COVID-19 pandemic.

The mean age of the participants was 50.4 (18.5) years: 48.6% were male and 19.9% self-reported as tobacco smokers (including recreational drugs). The most common presenting condition was suspected cardiac chest pain (66.0%). The median (range) COHb concentration was 0.9% (0–24.1%); 0.8% (0–6.0%) in self-report non-smokers and 2.2% (0–24.1%) in self-report smokers. Median concentrations were similar across presenting complaints (Table 2). The median (IQR) time between presentation at the ED and COHb measurement was 37 (25–56) minutes.

**Table 2:** Characteristics of participants by presenting condition

	All	Cardiac chest pain	Non-traumatic headache	Flu symptoms	Seizure	Syncope
Number of participants	4175	2755	545	81	99	695
Male, *n* (%)	2028 (48.6)	1470 (53.4)	175 (32.1)	32 (39.5)	47 (47.5)	304 (43.7)
Age (years) mean (SD)	50.4 (18.5)	51.7 (17.6)	43.0 (17.3)	45.7 (16.6)	41.2 (17.1)	53.2 (21.1)
Smokers, *n* (%)	831 (19.9)	554 (20.1)	120 (22.0)	18 (22.2)	30 (30.3)	109 (15.7)
						
COHb (%) – *n*, median, min, max	4175 0.9, 0, 24.1	2755 0.9, 0, 10.2	545 0.8, 0, 8.5	81 0.9, 0.2, 6.6	99 0.9, 0.3, 24.1	695 0.9, 0, 7.3
Non-smokers	3344 0.8, 0, 6.0	2201 0.8, 0, 6.0	425 0.8, 0, 2.4	63 0.8, 0.5, 1.8	69 0.8, 0.3, 1.8	586 0.9, 0, 5.3
Smokers	831 2.2, 0, 24.1	554 2.2, 0, 10.2	120 2.2, 0.3, 8.5	18 1.6, 0.2, 6.6	30 2.2, 0.6, 24.1	109 2.1, 0.6, 7.3

### Prevalence by case definition

The prevalence of raised COHb adjusted for smoking status in ‘suspected’ cases was 0.62% (95% confidence interval 0.41–0.91%) (Table 3). There was no association between cases of elevated COHb and presenting symptom, Pearson chi2(4) = 4.817; Pr = 0.307 (data not shown). Using the COMA screening tool, 1.05% (44/4175) respondents indicated that other people in the same property were affected (COMA_C); 1.65% (69/4175) indicated that their symptom improved when they were out of the property (COMA_O); and 2.16% of respondents (90/4175) answered ‘Yes’ to both COMA_C and COMA_O.

**Table 3:** Number of participants with raised COHb overall and by smoking status

Overall *n* (%, CI)	Non-smokers with COHb > 2% *n* (%, CI)	Smokers with COHb > 10% *n* (%, CI)
*n* = 26 0.62% (0.41%, 0.91%)	*n* = 23 0.69% (0.44%, 1.03%)	*n* = 3 0.36 (0.07%, 1.05%)

Of the suspected cases, 106 had a referral to the gas safety engineers (NGES) for an assessment of the home environment to detect for a possible source of CO. Data from gas engineers of ambient CO and gas appliance checks were linked to 52 of the participant’s homes. Where data were not able to be linked between hospital visit data and gas engineers, it was due to incomplete data or inability to identify the participant from the information in the gas engineer records. Engineers identified CO safety concerns in 22 homes (42.3%). There was 1 confirmed case of CO being present in the home from an ambient CO recording, and 21 ‘probable’ cases based on a report of a possible source of CO from a faulty or potentially faulty gas appliance in the home. Of these 22, 19 participants (90%) did not have raised COHb. Gas safety engineers identified 44 appliances that were a cause for concern in the 22 homes where there were confirmed or probable cases of CO exposure, which required further investigation to determine if each appliance was safe to use (the range of faulty appliances varied from between 1 and 3 per home).

### Evaluation of the COMA screening tool

This study evaluated the utility of the COMA screening tool against elevated COHb levels. There were 3240 participants with complete data for both COHb and the COMA screening questions excluding participants with missing responses or ‘did not know’ to COMA_M and COMA_A. Of the 26 subjects with elevated COHb, 3 responded ‘did not know’ to COMA_M and COMA_A. The COMA tool identified 11/21 (sensitivity = 52.4%) of subjects with elevated COHb. The positive predictive value was very low (0.9%) (Table 4).

**Table 4:** COMA positive and elevated COHb responses

	Elevated COHb		
COMA Positive	Yes	No	Total
Yes	11	1172	1183
No	10	2047	2057

## Discussion

### Main finding of this study

In this study, the investigators provide evidence of suspected, probable and confirmed unintentional CO exposure in participants presenting to EDs in the UK with symptoms suggestive of CO poisoning. The results show that participants attending the ED with symptoms that could be suspicious for CO exposure are at risk from CO poisoning from faulty appliances in the home. Studies that report the prevalence of occult CO exposure in ED do so based on abnormal CO levels (suspected cases) and do not link this to the presence of a source of CO exposure and therefore these cases are not confirmed. Direct comparison with other studies is difficult due to the heterogeneity in reporting methods and the CO thresholds used to denote exposure, with CO measured variously using blood gas COHb, pulse-CO oximetry and CO breath analyser devices.

### What is already known on this topic

Low-level unintentional carbon monoxide exposure is under-recognized by both participants and clinicians. The study found an actual or possible source of CO exposure in 22 homes that participants were unaware of. This has the potential consequence of long-term repeated exposure to harmful CO leading to symptoms lasting months or years, and progression to long-term health effects including neurocognitive sequelae and death.^21^ The potential for unintentional CO exposure in the home from faulty gas appliances has been previously investigated. Two studies of CO levels in UK homes found 18–23% exceeded the ambient World Health Organisation (WHO) guideline for safe levels.^22^^,^^23^ A study of 104 city homes found defective gas appliances in 34.6%.^24^ An association has been found in participants with self-reported neurological symptoms and risk of CO exposure caused by faulty gas appliances but without confirmation of exposure via COHb levels.^25^

### What this study adds

The strengths of this study include the large number of participants and the linking of CO environment data with the clinical presentation of the participant, using all components of the diagnostic triad of CO poisoning. This study reports the first limited evaluation of the COMA tool in clinical practice. Findings suggest the COMA_C and COMA_O questions in the COMA screening tool, may give a useful indication of CO exposure over and above an elevated COHb. The participant with a confirmed source of CO in the home, and 13 of the 21 probable cases of CO exposure (based on potentially faulty appliances), did not have a raised COHb, and NGES were contacted based only on their ‘yes’ to COMA_C and COMA_O responses. This challenges the current diagnostic triad of CO poisoning that includes the need for an elevated carboxyhaemoglobin level alongside symptoms consistent with CO poisoning and a history of recent CO exposure.^11^^,^^18^

The results of this study would suggest that the COMA tool could be used more widely in determining which patients should be advised to contact gas safety or public health agencies to investigate any possible environment exposure risk.

Carbon monoxide screening in undifferentiated patients brings a low yield of positive cases, which may make routine screening impractical to perform, with rates of less than 0.2% reported. The clinical symptoms of CO poisoning correlate poorly to COHb levels and therefore normal levels should not be used to rule out CO exposure.

### Limitations of this study

The lack of standard case definitions for CO exposure makes comparison across populations and studies difficult. In this study, a carboxyhaemoglobin threshold for diagnosing CO poisoning of > 2% in non-smokers and > 10% in smokers were chosen. Clinical guidance ranges vary internationally from those in this study and therefore COHb values may lead to an over- or under-estimation of suspected CO cases dependent on which guidance is used.

The study ended early due to the COVID-19 pandemic and did not achieve the initial recruitment target. In addition, 200 patients were excluded from the analysis due to a blood sampling error. Although this was under-recruitment against the initial target, this had no bearing on the findings which calculate prevalence in the recruited population.

The COHb levels reported in this study are likely to be an underestimation due to the COHb levels decreasing over time away from CO source.^9^ This is a factor when diagnosing CO exposure in the clinical environment. As this was a surveillance study on patients at the time of their presentation to ED, the study did not set out to estimate the COHb levels based on suspected time of exposure but it is known that the half-life of CO in the blood is around 4 hours in normal conditions reducing to around 80 minutes on 100% oxygen.^26^ Thus, measurements taken in the ED are lower than those that would have been present in patients at the peak of their exposure.

Although the yield for probable and confirmed cases in this study was low, the case definition was based on record linkages from gas engineers that were not available for all those referred. The data collected routinely by gas safety engineers during environment visits did not always provide clear detail to determine if faulty appliances or other sources were found, and, therefore, these cases were categorized as suspected.

## Conclusions

Unintentional carbon monoxide poisoning is a common and preventable condition that requires greater focus as a public health concern. Diagnosis of CO poisoning is essential in preventing further harm and the long-term consequences of exposure. Despite advances in gas and appliance safety this study provides evidence that unintentional CO exposure continues to pose a significant and unrecognized health burden. Greater emphasis should be given to simple preventative strategies, such as the installation and correct maintenance of fuel burning appliances and CO alarms.

The recommendation of this work is that the COMA guidance, in particular ‘Yes’ to COMA_C and/or COMA_O, is used in conjunction with testing of COHb levels at the earliest opportunity to ensure that participants with potential CO exposure are not missed. Further work is needed to confirm the utility of the COMA screening tool in differentiating those exposed to CO sources compared with those that are not. The most important factor in diagnosing CO exposure remains a high level of clinical suspicion by clinicians in primary and emergency care to the possibility of exposure in patients presenting with non-specific clinical symptoms.

## Supplementary Material

Supplementary_material_1_protocol_fdad007

Supplementary_material_2_full_inclusion_fdad007

## Data Availability

The datasets used and/or analysed during the current study are available from the corresponding author on reasonable request.

## Authors’ information

H.J. is a Visiting Reader at St George’s University of London and Visiting Professor Emergency at Cardiovascular and Critical Care Research Group, Kingston University. SP is a Visiting Professor at the University of Hertfordshire.

The authors would like to acknowledge Simon Clarke for his involvement in the study conception, and Phil Burrows and Andrew Morley for their contribution to the design, data collection and write-up of this work.

## Notes

**Heather Jarman**, Research Director for Nursing, Midwifery and Allied Health Professions

**Richard W. Atkinson**, Emeritus Professor of Epidemiology

**Desislava Baramova**, Clinical Research Practitioner

**Timothy W. Gant**, Head of Toxicology

**Tim Marczylo**, Principal Toxicologist

**Isabella Myers**, Consultant on Health, Policy and the Environment

**Shirley Price**, Emeritus Professor of Toxicology

**Tom Quinn**, Emeritus Professor of cardiovascular nursing
