# Supplementary material for: Screening patients for unintentional carbon monoxide exposure in the Emergency Department: a cross-sectional multi-centre study
Source: J Public Health (Oxf). 2023 Jan 31;45(3):553–9. doi: 10.1093/pubmed/fdad007 (PMC10470336; doi:10.1093/pubmed/fdad007)
Supplement: Supplementary_material_1_protocol_fdad007 [file supplementary_material_1_protocol_fdad007.pdf]

St George's University of London

St George's University Hospitals NHS Foundation Trust

Study Protocol

The ED-CO study: a prospective enhanced surveillance study of carboxyhaemoglobin (CO) levels in patients attending the Emergency Department with symptoms suggestive of CO exposure

ED CO

Screening for Carbon Monoxide Exposure in Emergency Departments

Sponsor's Project Number: 18.0052

REC Reference Number: 18/LO/1381 London – Queen Square

IRAS ID: 248936

Protocol Version and Date: v1.2 07/10/2019

Chief Investigator: Heather Jarman

Contents

1 List of abbreviations 3
2 Study synopsis 4
3 Study schema 6
4 Background 7
5 Research question 9
6 Study design9
7 Participation selection criteria 10
8 Participant recruitment process 11
9 Consent procedures 12
10 Study procedures 13
11 Methods 17
12 Data management and quality assurance 18
13 Archiving arrangements 19
14 Statistical design 19
15 Committees in involved in the study 23
16 Direct access to source data 23
17 Ethics and research governance requirements 24
18 Publication policy 25
19 Statement of compliance 27
20 List of protocol appendices 27
References 29
Appendix 1: definition of target populations 31
Appendix 2: detailed inclusion / exclusion criteria 33

1 List of abbreviations

ACS	Acute Coronary Syndrome
CI	Chief Investigator
CRF	Case Report Form
CO	Carbon Monoxide
COHb	Carboxyhaemoglobin
ED	Emergency Department
GCP	Good Clinical Practice
HPA	Health Protection Agency
HSE	Health and Safety Executive
ICF	Informed Consent Form
ISF	Investigator Site File
NGES	National Gas Emergency Service
OFGEM	The Office of Gas and Electricity Markets
PHE	Public Health England
PI	Principal Investigator
PIS	Participant Information Sheet
REC	Research Ethics Committee
SMF	Study Management File
SMG	Study Management Group
SOP	Standard Operating Procedure
SSC	Study Steering Committee
STEMI	ST Elevation Myocardial Infarction
WHO	World Health Organisation

2 Study synopsis

Brief title:	The ED-CO study: screening for carbon monoxide exposure in Emergency Departments
Official title:	The ED-CO study: a prospective enhanced surveillance study of carboxyhaemoglobin (CO) levels in patients attending the Emergency Department with symptoms suggestive of CO exposure.
Sponsor reference number:	18.0052
Public database identifier	The study will be registered on the ISRCTN database
Research question	What proportion of patients presenting to the Emergency Department with symptoms suggestive of carbon monoxide exposure have raised COHb levels?
Study design	Cohort observational
Study population / disease condition	Patients presenting to the study sites with the symptoms suggestive of carboxyhaemoglobin exposure
Eligibility criteria:	Inclusion criteria:<br>Patients aged 18 years or over presenting to the ED with symptoms suggestive of:<br>• Cardiac chest pain<br>• Non-traumatic headache<br>• Flu-like symptoms unless suggestive of specific focus of infection<br>• Seizures<br>• Syncope/presyncope
	Exclusion criteria:<br>• Patients under 18 years of age<br>• Chest pain associated with chest wall tenderness or non-cardiac cause (pulmonary embolism, pneumothorax)<br>• Recurrent situational syncope<br>• Head injury<br>• Actual or suspected smoke inhalation<br>• Patients unable to understand the informed consent process and/or has a poor understanding of English (e.g. English-speaking relative/translator not available within timescales for study procedures)<br>• Patients previously enrolled in this study
Target number of participants	5222
Anticipated start date	01 September 2018
Anticipated end date	30 April 2020

Primary aim	To determine, in patient presenting to the EDs with symptoms suggestive of raised carboxyhaemoglobin levels, the proportion with high COHb levels that could be caused by CO exposure from domestic or occupational source.
Secondary aim(s)	• To validate the 'COMA' acronym screening questions for identification of patients with elevated COHb in patients presenting with symptoms of CO exposure<br>• To determine if there is seasonal variation in the levels of CO exposure<br>• To derive and validate the optimal algorithm for point of care carboxyhaemoglobin testing for identification of patients with symptoms of CO exposure<br>• To determine if a novel biomarker for CO exposure can be identified<br>• To estimate the frequency of a recorded diagnosis of dementia in patients with and without elevated COHb levels

3 Study schema

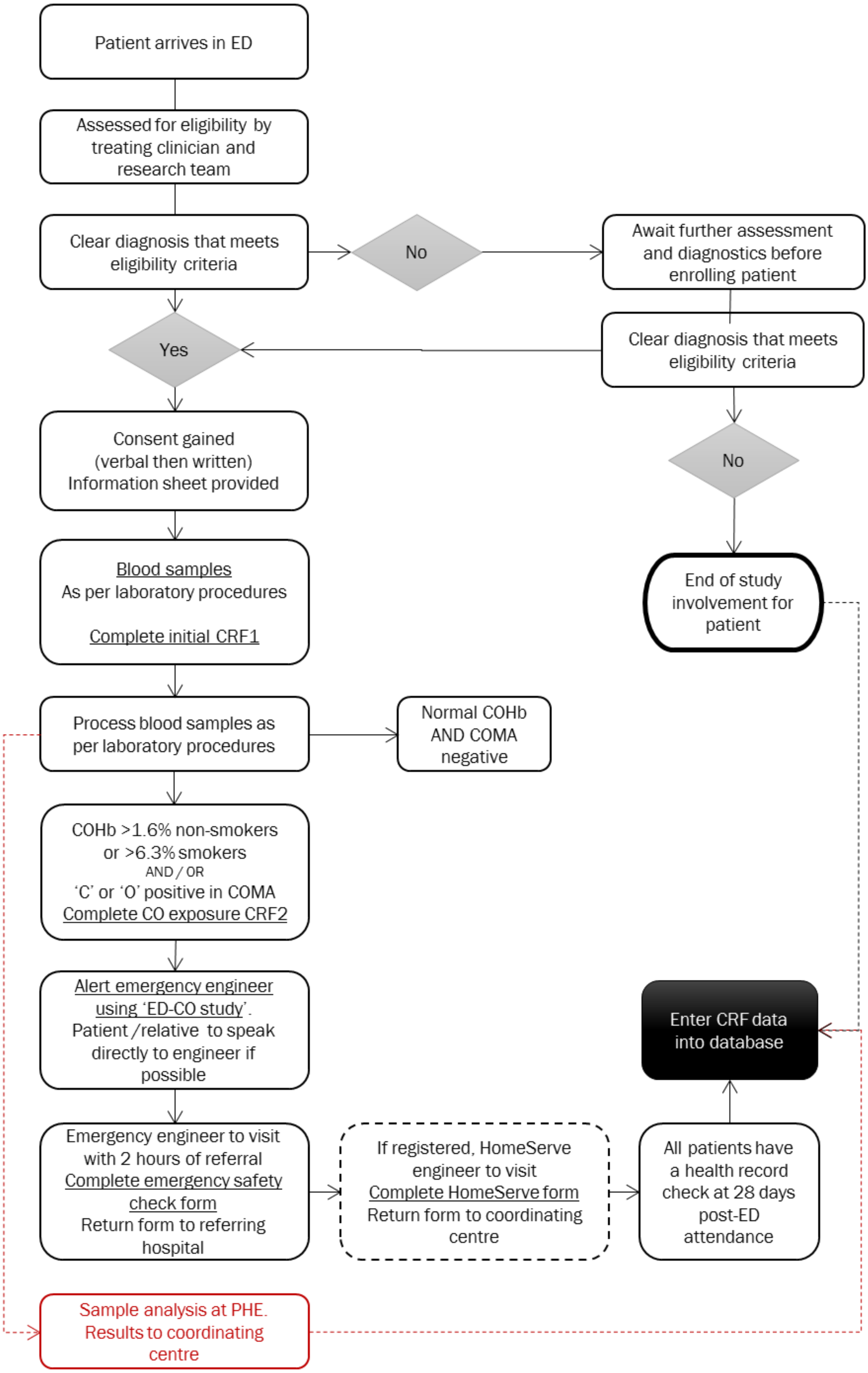

4 Background

4.1 Study rationale

Carbon monoxide is reported to cause around 30 deaths [1], 200 admissions [2] and 4000 presentations to Emergency Departments (EDs) each year in the UK [3]; however, these figures are estimates and the true prevalence of CO poisoning is not known. Studies published in the U.S. and Malta suggested that the prevalence of CO poisoning is low in an undifferentiated healthcare population [4-7] but much higher in targeted groups (those showing clinical features that can be caused by CO, such as non-traumatic headache, symptoms suggestive of cardiac chest pain, exacerbation of chronic lung disease, flu-like symptoms and seizures) [8-14]. The incidence of neurocognitive effects following CO poisoning is unknown, although reported in the literature [15].

Both acute and chronic carbon monoxide (CO) exposure can produce a wide variety of non-specific clinical features, all of which can mimic other pathologies. There is evidence to suggest that CO toxicity is frequently missed by healthcare professionals [16] and those who continue to be exposed to CO may experience chronic neurocognitive dysfunction [17, 18].

Exposure to CO is responsible for the formation of carboxyhaemoglobin (COHb) where CO replaces oxygen bound to haemoglobin in red blood cells. Similarly, CO can bind to heme groups in mitochondria. Thus oxygen depletion and inhibition of oxygen use in respiration are the most likely mechanism for the symptoms of CO poisoning. As such, blood COHb level is a recognised biomarker for CO exposure. In England a study published in 2012 showed unexpectedly high carboxyhaemoglobin (COHb) levels in between 2.1% - 7.5% of patients presenting to four large EDs with chest pain, exacerbation of chronic obstructive pulmonary disease, non-traumatic headache, flu-like symptoms or flu-like illness [19]. The actual prevalence of CO exposure could not be confirmed because systematic investigation of possible domestic or occupational sources of CO was not undertaken. In addition, identification of smoking as a confounder relied on self-reporting by patients on a health questionnaire but this was found to be unreliable with some patients being reluctant to admit that they smoked. Smoking is a known source of chronic CO exposure and the baseline level of COHb in smokers is significantly higher than in non-smokers.

A study of ambient CO levels in 597 homes in Greater London and South East England found that 22% had at least one defective gas appliance that was deemed to be unsafe and 7% of the 1414 appliances that were tested were reported to be immediately unsafe [20]. This showed a remarkable degree of agreement with 2 previous surveys undertaken by the same authors which found that 23% of 56 homes and 18% of 270 dwellings tested respectively exceeded WHO guidelines for ambient CO levels (which at that time were the only health-based guideline levels available). Although a brief health questionnaire of householders hinted at a correlation between defective gas appliances and symptoms, the studies did not

take into account the effect of potential confounders such as smoking habits and occupation. Unfortunately, these studies do not allow a direct link to be made between domestic ambient concentrations that are higher than WHO ambient guideline levels and either raised COHb levels or adverse health consequences.

A problem associated with the use of COHb as a biomarker of CO exposure is the relatively short half-life in blood of 4-6 hours. This is especially influential in the ED as the half-life of blood COHb can decrease faster when oxygen is administered prior to the levels being measured. As such, levels of COHb may be significantly reduced in some individuals attending EDs, the level of reduction dependent upon time since last exposure and use of oxygen therapy. This reduction in COHb levels and any related improvement in symptoms can result in a clinicians' level of suspicion of CO poisoning in patients attending EDs being reduced which can result in misdiagnosis. Clarke et al 2012 [19] found a mean COHb level of 8.7% in patients presenting to the ED with headache. This level is lower than the threshold usually used by clinicians of greater than 10% as an indicator of CO poisoning. Overall 56% of patients in this study with confirmed sources of CO exposure had COHb below 10%.

Lastly, two studies from Taiwan have suggested that carbon monoxide exposure may be linked with dementia [21, 22] and the guidance produced by the Department of Health [23] identifies that clinical staff should be aware that prolonged exposure to CO may have lasting neurological effects. Such an association has not been established in the U.K.

The acronym 'COMA' was developed to prompt clinicians to consider CO exposure in their list of differential diagnoses [23]. When a patient presents with a symptom that could be caused by CO exposure the clinician is advised to ask the following questions:

- **C**: Cohabitees/companions. Is anyone else in the property affected (including pets)? Y/N
- **O**: Outdoors. Do your symptoms improve when out of the building? ('better outdoors') Y/N
- **M**: Maintenance. Are your fuel-burning appliances and vents properly maintained? Y/N
- **A**: Alarm. Do you have a carbon monoxide alarm? Y/N

If the patient answers yes to either of the first 2 questions or no to either of the last 2 questions, then the clinician is prompted to consider CO exposure and take appropriate clinical and public health measures. Public Health England has produced a guideline to this effect [23] but this has not been validated.

5 Research question

This study aims to answer the question: “What proportion of patients presenting to the Emergency Department with symptoms suggestive of carbon monoxide exposure have raised carboxyhaemoglobin levels?”

5.1 Primary aim

The primary aim is to determine, in patients presenting to the EDs with symptoms suggestive of raised carboxyhaemoglobin levels, the proportion with high COHb levels that could be caused by CO exposure from domestic or occupational source.

5.2 Secondary aim(s)

- To validate the ‘COMA’ acronym screening questions for identification of patients with elevated COHb in patients presenting with symptoms of CO exposure
- To determine if there is seasonal variation in the levels of CO exposure
- To derive and validate the optimal algorithm for point of care carboxyhaemoglobin testing for identification of patients with symptoms of CO exposure
- To determine if a novel biomarker for CO exposure can be identified
- To estimate the frequency of a recorded diagnosis of dementia in patients with and without elevated COHb levels

6 Study design

The design of this study has been adopted following discussions in the study management committee.

6.1 Methods

A prospective enhanced surveillance study of COHb levels in patients attending participating EDs with symptoms suggestive of CO exposure will be undertaken.

The research comprises a number of inter-related elements using data as follows:

- Routine blood sample analysis and clinical data relating to the presentation. Additional detailed questions will be applied in patients with a raised COHb or those with possible CO exposure identified using the ‘COMA’ questions
- Anonymised blood sample analysis performed for validation of a point of care test system and cotinine levels by Public Health England (PHE) laboratories

- Linked environment / CO data from the scene of suspected CO source for patients with confirmed or suspected CO exposure and a sample of patients without suspected CO source/exposure (to evaluate COMA questionnaire)

- Routine clinical data for identification of patients with a formal diagnosis of dementia in patients with possible CO exposure

7 Participation selection criteria

Patients presenting to the study site Emergency Department with symptoms suggestive of carbon monoxide exposure will be screened according to the inclusion and exclusion criteria for the study. Participants will be considered eligible for enrolment into this study if they fulfil all of the inclusion criteria and none of the exclusion criteria as defined below. A detailed list of criteria can be found in Appendix 2. Eligible participants will be entered onto the *participant screening and enrolment log* and assigned a study specific identification number.

Notices will be placed in the assessment (triage) area of ED explaining that the study is taking place.

7.1 Inclusion criteria

Patients aged 18 years or over presenting to the ED with symptoms suggestive of:

- Cardiac chest pain
- Non-traumatic headache
- Flu-like symptoms unless suggestive of specific focus of infection
- Seizures
- Syncope / presyncope

7.2 Exclusion criteria

- Patients under the age of 18 years
- Chest pain associated with chest wall tenderness or non-cardiac cause (pulmonary embolism, pneumothorax)
- Recurrent situational syncope
- Head injury
- Actual or suspected smoke inhalation
- Patients unable to understand the informed consent process and/or has a poor understanding of English (e.g. English-speaking relative/translator not available within timescales for study procedures)
- Patients previously enrolled in this study

8 Participant recruitment process

Patient recruitment at a site will only commence once evidence of the following approval / essential documents are in place:

1. REC approval, if applicable

2. Final sponsorship and host site permissions,

All patients who wish to enter the study will be fully screened and consented by the Chief Investigator, or an appropriate delegate as detailed in section 9.1.

Patient enrolment will be conducted over a 12 month period across the two hospital sites. The study incorporates an internal pilot phase covering 3 months during the first months of recruitment. Recruitment will not stop after the pilot phase but the SSC will be asked to review recruitment rates relative to the overall target. They will give consideration to the need for the study to capture more patients over a winter period when the use of appliances that may emit CO (i.e. boilers and heaters) is more prevalent and make recommendations of the need for specific or variable recruitment rates over the duration of the study.

Patients will be identified at the time of arrival by clinical staff and / or study trained research staff from their initial ED assessment ('triage'). Patients eligible for the study that agree to participate will be assessed clinically in the ED according to standard practice for the site. All eligible patients will be undergo blood sampling within 30 minutes of eligibility being confirmed and have baseline information recorded on the ED screening CRF. If the patient's blood CO level is normal and the answers to the COMA questions are negative then there is no further involvement for the patient.

If the CO level is raised or the patient answers the 'COMA' questions in the affirmative then they will be asked for further information regarding potential CO exposure. An emergency gas engineer will be contacted, as is routine practice for patients with suspected CO exposure, to investigate the scene of a suspected exposure for all patients with raised COHb and / or clinical suspicion of CO exposure. Clinical suspicion may be based on responses to the 'COMA' questions or the treating clinician's concerns. The scene of expected exposure may be either the patient's own home, another home or their workplace.

Following their initial baseline visit patients will not be required to attend the hospital at any other times for the purposes of the study. The total study duration is estimated to be approximately 18 months to allow for final data clean-up, processing and analysis.

9 Consent procedures

9.1 Informed consent

9.1.1 Patients with capacity

Informed consent will be obtained by the local Principal Investigator (PI), or an appropriately trained member of the team, as a two-part process prior to inclusion into the study. To reduce delays to routine sampling of blood during the triage process in the ED, verbal consent will be obtained following eligibility assessment in order for the initial blood samples to be taken.

If the patient declines to give verbal consent at this stage they may be given further written information and the patient given time to consider their participation.

If verbal consent is used initially then patients will subsequently be given a written patient information sheet describing the study and the study team will be available to answer any questions. The patient will be given time to read the information and consider their participation in the study. If they still wish to take part they will then go through the written consent process. This will be deferred written consent for the blood sampling and written consent for the other aspects of the study. If they choose to end their participation at this point then any blood samples will be discarded.

For some patients there may be uncertainty about the diagnosis until further investigations and assessment have been made in the ED. To avoid unnecessary blood sampling in this group, study eligibility assessment and consent may be delayed until a clear diagnosis it made. This group will have written consent obtained prior to blood samples being taken.

The patient will be informed that their medical records are subject to review by representatives of the sponsor as necessary and that data will be collected and processed in accordance with the Data Protection Act 2018. The patient will be told that participation in the study is voluntary and that they are free to withdraw from the study at any time and without prejudice. If patients are willing to provide a reason for their withdrawal, this information will be recorded. Each patient will be advised that data collected may be published or presented at scientific meetings and may also be subject to audit procedures from Regulatory Authorities. All such personally identifiable data will be pseudoanonymised to maintain patient confidentiality.

A copy of the signed Informed Consent Form (ICF) along with a copy of the most recent approved Patient Information Sheet (PIS) will be given to the study participant. The original signed & dated consent form will be placed in the ISF and a copy retained in the medical notes.

If new information results in significant changes to the risk–benefit assessment, the consent form will be reviewed and updated if necessary. All participants, including those already being treated, will be informed of the new information, given a copy of the revised consent form and asked to re-consent if they choose to continue in the study.

9.1.2 Patients who lack capacity

Some patients eligible for the study may have impaired capacity at the time of attendance due to altered conscious levels or ongoing cognitive impairment (for example, dementia). This group is an important cohort. If a consultee is available within the timeframe for procedures (i.e within 30 minutes of eligibility confirmation) then consultee approval will be sought.

If and when patients regain the physical and mental capacity to give consent, information will be provided to them and written informed consent will be sought for continuation in the study. If a patient declines to give consent for continuation at this stage, their wishes will be respected. All samples and paperwork relating to the study will be destroyed.

9.1.3 Definition of the end of study

The end of study is defined as the last patient recruited + 28 days to allow for follow-up completion.

The REC requires notification of the end of study within 90 days of its planned completion or within 15 days if the study is terminated early. The sponsor will facilitate assistance and compliance with requirements.

10 Study procedures

Each site will have a nominated study lead. Research staff from within the local clinical team will complete all study procedures.

10.1 Screening assessments

All patients attending the Emergency Department will undergo routine clinical assessment including clinical judgment, blood test measurement and further investigations as per standard practice at the hospital. This information will be used to determine if patients meet the study eligibility criteria.

10.2 Baseline assessments

10.2.1 Blood samples

All patients considered eligible for the study will have blood drawn for analysis for COHb, haematocrit, cotinine and biomarkers. Cotinine is the main metabolite of nicotine, and its

measurement offers higher accuracy than self-reports of tobacco smoking, and will be used to assess the smoking status of patients.

Eligible participants will be consented as laid out in section 9.1. Blood samples are a standard venous blood gas sample and haematocrit, which would be taken for routine clinical purposes in this group of patients, and two additional 4ml samples for study purposes. Study samples will be processed and sent to the Public Health England laboratory to test a proposed new point-of-care system for carboxyhaemoglobin (all patients) and to test cotinine levels (in patients with high COHb levels) to determine their smoking status.

The small volume of blood drawn for research purposes (approximately 8 - 10ml or two teaspoons) will not cause any harm to participants, who are already undergoing a blood test. This sample will be discarded in the event that participants decide not to participate in the study. The approach described has been taken in other studies with a similar rationale where there is a time factor in taking samples.

Research blood sample bottles will be labelled with a unique study identifier and processed according to study laboratory procedures.

10.2.2 Case Report Form (ED attendance)

Following full written consent being obtained all eligible patients will have the CRF completed. Information on age, gender and ethnicity will be collected. This data is collected as part of routine NHS admission procedures and is not specific to the study. The following study specific information will be recorded:

- Onset of symptoms
- COMA screening tool questions
- Brief medical history
- Smoking status
- Possible CO exposure sources

Patients who have a raised COHb level and those who answer yes to 'C' or 'O' COMA questions or no to 'M' or 'A' questions will be asked additional questions about their likelihood and possible cause of CO exposure.

Patients who have their COHb levels measured but who do not complete the detailed CRF (either for medical or logistical reasons) will be contacted at a later date. This may be on the ward, if the patient has been admitted, or by telephone, if the patient has been discharged. It is very important to contact this latter group, not only to try to complete the questionnaire, but to check that appropriate remedial action has been taken to remove the source of CO exposure.

10.2.3 Immediate investigation of scene – emergency gas safety check

Patients who are considered as being ‘positive’ for exposure to CO (raised COHb level > 1.6% in non-smokers and > 6.3% in smokers) on venous gas sampling, and / or those who answer positively to the ‘C’ or ‘O’ questions of the COMA acronym will be referred to the National Gas Emergency Service (NGES) engineers using standardised Health and Safety Executive guidance for reporting of a potential CO exposure.

The NGES will undertake an investigation of the scene (either the patient’s home or work premises dependent on potential exposure source identified), take actions to control any identified hazard and liaise with the HSE for possible occupational sources. This is standard practice for hospitals identifying possible CO exposure incidents.

With patient consent a member of the research team will contact the identified emergency referral number to make the initial referral and then allow the patient to speak directly to the engineer. This referral by the research team will identify the patient as an ‘ED-CO study referral’ so that the engineers are clear of the need to return a copy of the standard report form to the referring hospital.

If the patient lacks capacity to consent, or in circumstances where the engineers cannot access the property to carry out a full inspection, an investigation of the site can still be undertaken. This is standard practice for hospitals identifying possible CO exposure incidents.

The timing of the call to the gas engineers is important as gas safety engineers are required to investigate the scene and make appliances (irrespective of fuel type) safe within 2 hours of the notification and will require access to the property. This referral should be made as soon as possible CO exposure is identified to ensure that the risk to others who may be affected at the scene is reduced. Gas safety engineers visiting the scene are registered as trained to gas safety standards (see normative documents: https://www.euskills.co.uk/about/our-industries/gas/standards-setting-body/

If a dangerous appliance is found, National Gas Emergency Service engineers will disconnect the appliance if immediately dangerous. If not immediately dangerous, the gas engineer will ‘make safe’ the appliance and tell the occupant to contact their normal service provider (e.g. British Gas, HomeServe).

An emergency safety check form will be completed by the engineer based on this visit and a copy sent to the referring hospital for upload of study relevant data, including ambient CO levels on to the study database.

10.2.4 Gas appliance safety check

Following the initial emergency safety check visit it is standard practice that a follow-up visit is arranged with a gas engineer to check and then repair or replace dangerous appliances. If indicated, the patient will be advised to contact their usual service engineer to rectify problem appliances. If the occupant does not have a regular service engineer information will be provided to ensure that a Gas Safe Registered engineer can be contacted to undertake the work. Helpline numbers will be provided for identifying registered oil and solid fuel engineers. HomeServe have agreed that if they are contacted, they will offer a free appliance check and properties without a CO alarm will be provided with a free alarm and information on fitting. Recertification of appliances requiring parts or further visits will be undertaken at the expense of the patient, who will be advised of this prior to any work being undertaken. Patients who are eligible for free appliance maintenance according to the OFGEM priorities register, will have their further servicing needs covered.

If the patient's (owner's) appliance checks are carried out by HomeServe then HomeServe will return information relating to the visit up to the completion of the free appliance check to the coordinating centre using secure email and the patient's unique study ID. If HomeServe is not the usual provider, then no further data for the study will be provided. Follow-up data for dementia diagnosis

There is evidence of patients exposed to carbon monoxide having neurocognitive problems that manifest after initial poisoning (up to one month post exposure ceasing), or a continuation of symptoms that are still present by one month following exposure ceasing. The research team will review the ED notes and hospital discharge codes (for those patients who were admitted as in-patients) of all recruited patients at 28 days post-attendance for a formal diagnosis of dementia if this is not known at the time of admission. This will be recorded on CRF2 (possible exposure).

10.3 Public health concerns

It is imperative that all patients who are deemed to have abnormally high COHb levels are followed up. Clinicians have a duty to inform Public Health England where there is a potential risk to public health, for example a risk to other household members. The local Public Health England Centre (PHEC) needs to be contacted as soon as the diagnosis is made if the source is thought to be domestic or occupational. Contact details for local PHEC's can be found at https://www.gov.uk/guidance/contacts-phe-regions-and-local-centres#centres. The PHEC's role is to assess the public health issues and ensure action is taken to protect others at risk and prevent recurrence. In particular, it is essential that faulty appliances are rendered safe as rapidly as possible. This is done in partnership with other involved agencies (HomeServe and the National Gas Emergency Service). Further information on

management of CO incidents is found on the PHE web-site:
https://www.gov.uk/government/collections/carbon-monoxide-co

10.4 Summary flow chart of study assessments

	Day 1	Day 1	Day not specified	28-day follow-up
	Prior to full written consent	Following full written consent		
Perform routine initial assessment / study screening	X			
Check eligibility	X			
Provide patient information leaflet if applicable	X			
Obtain verbal or written consent for blood sampling	X			
Collect blood for CO levels (venous gas)	X			
Collect blood for point of care validation and cotinine (ETDA and SST samples)	X			
Obtain full written consent if not already	X			
Complete initial CO exposure screening CRF1 (Emergency Department)		X		
Refer for gas safety check if deemed positive for CO exposure		X		
Complete CO exposure suspected CRF2 (Emergency Department)		X		
Gas Safety check and completion of standard report form (if applicable)		X		
Homeserve check and completion of standard report form (if applicable)			X	
Establish dementia diagnosis status (if not known already)				X

Table 1: schedule of study assessments

11 Methods

11.1 Laboratory procedures

The time of blood draw and venous COHb result will be recorded in the case report form. The overall schedule for sampling blood is in outlined in Table 2: blood draw schedule. The venous gas sample will be processed in real-time using the department's blood gas machine. Blood drawn for laboratory analysis by PHE will be processed according to the laboratory procedures and stored in vials labelled with the patient's unique study number.

Time point	Sampling requirements	Number of samples for storage
Admission (T0)<br>Collected as soon as possible after presentation	1. Venous blood gas as part standard of care<br>2. ETDA (purple) x1 for haematocrit level as part standard of care<br>3. ETDA (purple) x1 (4ml)<br>4. SST tube (gold or yellow) x1 (4ml)	x1 whole blood in ETDA tube<br>x2 serum in cryovial polypropylene tube

Table 2: blood draw schedule

Following processing and storage the cyrovials will be collected weekly by specialist courier and transferred to the Public Health England laboratory for analysis of:

a. Cotinine levels using LC-orbitrap-MS using an in-house method to confirm patient smoking status

b. New methods of measuring carboxyhaemoglobin:

i. indirect quantification using a head-space GC-MS method for determining CO levels

ii. direct quantification using an MS method for intact CO-Hb

c. New biomarkers of CO exposure:

i. lipidome (UHPLC-orbitrap-MS)

ii. metabolome (UHPLC-orbiTrap-MS and ion chromatography MS)

Upon completion of sample analysis, remaining sample material will be disposed in accordance with the Human Tissue Authority's Code of Practice.

12 Data management and quality assurance

12.1 Confidentiality

All data will be handled in accordance with the Data Protection Act 2018.

Once written informed consent has been obtained for study participation, the patient will be allocated a unique study identifier (consisting of a site number and sequential participant number) which will be utilised to code all depersonalised data. The patient's unique study number (ID) only, will be used for identification.

Data generated from the study will not be shared outside of the UK.

12.2 Data collection tool

Paper Case Report Forms (CRFs) will be used. All data will be entered legibly in black ink with a ball-point pen. If the investigator makes an error, it will be crossed through with a single line in such a way to ensure that the original entry can still be read. The correct entry will

then be clearly inserted. The amendment will be initialled and dated by the person making the correction immediately. Overwriting or use of correction fluid will not be permitted.

It is the investigator's responsibility to ensure the accuracy of all data entered and recorded in the CRFs. The *delegation of responsibilities log* will identify all study personnel responsible for data collection, entry, handling and managing the database.

The source documentation is the CRF.

12.3 Data handling and analysis

Data will be uploaded, managed and stored on a password protected electronic database (RedCap) accessible only to members of the research team. No patient identifiable information will be recorded on this database. The patient's unique study number (ID) only, will be used for identification on study specific data.

Data collected in the ED on the Case Report Forms (CRF) will be held by each research site and entered as soon as possible into the study database by the local research team. Hard copies of theCRFs will be kept securely at each site.

The results of the gas safety engineer and Homeserve visits will be recorded on standard gas engineer forms. These contain patient identifiable information and will be emailed via secure server to the referring hospital and entered in to the database.

The results of the blood samples tested at the Public Health England laboratory will be emailed via secure server to the referring hospital and entered in to the database. These contain the patient's unique study number (ID) only.

Study specific paper CRFs will be stored in a secure location and kept until the end of the study and for up to 10 years to ensure that the source documentation is available for any query resolution.

13 Archiving arrangements

The essential study documents along with the study database will be archived in accordance with the sponsor requirements. The agreed archiving period for this study will be 10 years.

Each PI at any participating site will archive the study essential documents generated at the site for the agreed archiving period in accordance with the signed clinical study site agreement / statement of activities.

14 Statistical design

14.1 Statistical input in study design

Statistical input for this study has been provided by:

Professor Richard Atkinson, Professor of Epidemiology, Population Health Research Institute, St George's, University of London, Cranmer Terrace, London SW17 0RE.

Prof Simon Jones, Research Professor in Population Health, Center for Healthcare Innovation and Delivery Science, Department of Population Health, NYU School of Medicine, NYU Translational Research Building, 227 East 30th Street, New York, NY 10016.

14.2 Endpoints

14.2.1 Primary aim

The primary aim is to determine in patients presenting to EDs with symptoms suggestive of raised COHb levels the proportion of patients with high COHb levels that could be caused by CO exposure from domestic or occupational sources.

Adapted from Clarke et al 2012 [19] and Mandal et al 2011 [24], patients with COHb levels >1.6% (non-smokers) and >6.3% (smokers) and a confirmed environmental source of CO will be identified as confirmed cases. Smoking status will be determined from concordant responses to Q5 on the questionnaire and cotinine measurement. COHb measurements will be made using blood samples taken within 30 minutes of study eligibility being established. Confirmation or otherwise of an environmental source will be determined from the Gas Safety report following site visit by NGES. Patients with COHb levels >1.6% (non-smokers) and >6.3% (smokers) but without a confirmed environmental source of CO will be identified as probable cases. Confirmed and probable cases will also be combined and classified as positive cases. Patients with COHb levels <1.6% (non-smokers) and <6.3% (smokers) will be classified as negative cases.

This analysis will be repeated for each symptom set defined in the inclusion criteria i.e. cardiac chest pain; non-traumatic headache; flu-like symptoms; seizures; and syncope/pre-syncope.

14.3 Secondary aims

- To validate the 'COMA' acronym screening questions for identification of patients with elevated COHb in patients presenting with symptoms of CO exposure
- The endpoints for this aim will be number of positive (confirmed/probable) and negative case patients together with responses to COMA questions. To determine if there is seasonal variation in the levels of CO exposure

The proportion of positive cases will be tabulated by month/quarter.

- To derive and validate the optimal algorithm for point of care carboxyhaemoglobin testing for identification of patients with symptoms of CO exposure
- To determine if a novel biomarker for CO exposure can be identified
- To estimate the frequency of a recorded diagnosis of dementia in patients with and without elevated COHb levels

14.4 Sample size and recruitment

14.4.1 Sample size calculation

Clarke et al 2012 [19] estimated the prevalence of unexpectedly high COHb in an English ED population to be 4.3%. Based upon the attendance rates observed in this study and the recruitment rate (approximately 40%) we estimate a sample of 5222 over 15 months is required to identify 225 positive (confirmed or probable, i.e. raised COHb with or without CO source identified) cases.

As in Clarke et al., the prevalence of positive cases in was 4.3%. and can be expected to vary between disease groups, for example 17% of COPD cases and 3% of unstable angina cases have been found to be exposed to CO respectively, as measured by raised COHb levels [8, 10]. Therefore different disease groups must be considered separately when estimating the sample size.

The following formula, and associated binomial exact confidence interval, can be used to determine suitable sample sizes for the estimation of prevalence of confirmed/positive cases:

$$n = z2 *p(1-p)/d2$$

Where n = sample size, z = standard normal deviate (1.96 for a 95% confidence interval), p = estimated prevalence of raised COHb (from literature), d = level of accuracy desired / one-half the width of the confidence interval.

The above calculation returned samples size estimates of between 76 and 300 according to symptom group and desired precision.

The proportion of confirmed (*vs.* probable) cases is also likely to vary by symptom classification. For example, Clarke et al. 2012 [19] reported that from 22 patients presenting with headaches and raised COHb, 17 were classified as having confirmed CO exposure whereas for chest pain the proportion was much lower, 4 from 30 with confirmed CO exposure. Prevalence estimates for confirmed cases only will require larger sample sizes.

A secondary aim of the study is to evaluate the COMA questions. No previous evaluation of COMA has been undertaken to guide selection of sensitivity/specificity for sample size estimation. However, we have assumed that for the test to be viable in a clinical setting it

should have high sensitivity as it is important not to miss-diagnose a patient with high COHb. We are less concerned by the rate of false positives as the clinical consequences are minimal. Assuming the prevalence of positive cases to be 4.3% [19] and the desired sensitivity to be 80% then the number of patients needed to evaluate the COMA questionnaire would be over 1,230 (positive and negative cases) [25]. The sample size required to evaluate the sensitivity for confirmed cases only will be higher.

A further secondary aim of the study is to estimate the proportion of patients with a recorded diagnosis of dementia in positive and negative COHb cases. Assuming an age adjusted prevalence of dementia of 6.4% [26] and taking the estimated number of positive cases as 225 patients (assuming recruitment of 5222 patients) we will be able to estimate the prevalence with a 95% (binomial exact) confidence interval of 3.6%-10%. At present we have no information to estimate the expected difference in prevalence of dementia between positive and negative cases. We therefore propose the same sample size of 225 for non-cases (negative COHb and negative COMA) matched by age and gender.

14.5 Statistical analysis plan

14.5.1 Summary of baseline data and flow of patients

The flow of eligible patients is shown in Section 15.2.1

14.5.2 Primary aim analysis

The primary aim is to determine, in patients presenting to the EDs with symptoms suggestive of raised carboxyhaemoglobin levels, the proportion with high COHb levels that could be caused by CO exposure from domestic or occupational source.

A simple calculation will be carried out to estimate the proportion of confirmed cases in each disease group, and a 95% confidence interval will be calculated for each proportion. These calculations will also be repeated for positive cases.

Missing data: data for those patients who refuse consent will be collected, including age, sex, ethnicity, and disease group. The socio-demographic characteristics of the patients who refuse to consent will be compared with those of the recruited patients to check for evidence of selection bias.

14.5.3 Secondary aims analysis

- To validate the COMA acronym screening questions for identification of patients with symptoms of CO exposure

To validate the COMA screening questions for identification of patients with elevated COHb, a 2x2 contingency table will be constructed as follows (where Xi i=1-4 represent patient numbers in each category):

	Positive case	Negative case
COMA +ve	X1	X3
COMA –ve	X2	X4

The sensitivity, specificity and positive predictive values for the COMA questionnaire will be calculated using the standard methods.

This analysis will be repeated for each symptom set defined in the inclusion criteria i.e. cardiac chest pain; non-traumatic headache; flu-like symptoms; seizures; and syncope/pre-syncope.

- To estimate the frequency of a recorded diagnosis of dementia in patients in both the exposed and non-exposed groups

The proportion of patients with a recorded diagnosis of dementia (Section 12.2.2) by positive / negative case definition will be determined.

14.6 Interim analysis

No interim analyses are planned.

15 Committees in involved in the study

Study Management Group (SMG) - responsible for the day-to-day management of the study. The role of the group is to monitor all aspects of the conduct and progress of the study, ensure that the protocol is adhered to and take appropriate action to safeguard participants and the quality of the study itself.

Study Steering Committee (SSC) - provides overall supervision of the study and ensures that it is being conducted in accordance with the relevant regulations. The Study Steering Committee has agreed the study protocol and any protocol amendments and provides advice to the investigators on all aspects of the study.

16 Direct access to source data

The investigator(s)/institution(s) will permit sponsor / study-related monitoring, audits, REC review, and regulatory inspection(s), providing direct access to source data/documents.

Study participants are informed of this during the informed consent discussion. Participants will consent to provide access to their medical notes.

17 Ethics and research governance requirements

The site will conduct the study in compliance with the protocol as agreed by the Sponsor and which was given favourable opinion by the Research Ethics Committee (REC), Health Research Authority (HRA) and confirmation of local capacity and capability from site R&D.

The Chief Investigator will be provided (via the Sponsor) with file indexes. The CI will be responsible for the maintenance of the TMF and delegates the responsibility of ISF file maintenance to the PI at each participating site.

It is the responsibility of the Principal Investigator at each site to ensure that all subsequent amendments gain the necessary approval.

Within 90 days after the end of the study, the CI and Sponsor will ensure that the REC is notified that the study has finished by completing the Sponsor's 'End of study declaration'.

The CI will supply an End of Study report of the clinical study to the REC within one year after the end of the study.

17.1 Notification of serious breaches of GCP and/or the protocol

Any protocol deviations or violations will be documented using a protocol deviation form. No serious safety breaches are anticipated as this is a data only study.

A "serious breach" is a breach which is likely to effect to a significant degree:

(a) The safety or physical or mental integrity of the participants of the study; or

(b) The scientific value of the study.

The CI will notify the Sponsor immediately of any case where there exists a possible occurrence of a serious breach

17.2 Insurance and indemnity

St George's University Hospitals NHS Foundation Trust is the study sponsor and is covered by the NHS Resolution (previously the NHS Litigation Authority). NHS bodies are liable for clinical negligence and other negligent harm to individuals covered by their duty of care. NHS Institutions employing researchers are liable for negligent harm caused by the design of studies they initiate.

17.3 IP and development policy

Unless otherwise specified in agreements, the following guidelines shall apply: All Intellectual Property Rights and Know How (IP) related to the Protocol and the Study are and shall remain the property of the Sponsor excluding

1) Pre-existing IP related to clinical procedures of any Hospital.

2) Pre-existing IP related to analytical procedures of any external laboratory.

All contributors:

shall assign their its rights in relation to all Intellectual Property Rights and in all Know How, not excluded above to the Sponsor and at the request and expense of the Sponsor, shall execute all such documents and do all such other acts as the Sponsor may reasonably require in order to vest fully and effectively all such Intellectual Property Rights and Know How in the Sponsor or its nominee.

shall promptly disclose to the Sponsor any Know How generated pursuant to this Protocol and not excluded above and undertake treat such Know How as confidential information jointly owned between it and the Sponsor

Nothing in this section shall be construed so as to prevent or hinder and medical professional from using Know How gained during the performance of the Study in the furtherance of its normal business activities, to the extent such use does not result in the disclosure or misuse of Confidential Information or the infringement of any Intellectual Property Right of the Sponsor.

18 Publication policy

Publication: "Any activity that discloses, outside of the circle of study investigators, any final or interim data or results of the Study, or any details of the Study methodology that have not been made public by the Sponsor including, for example, presentations at symposia, national or regional professional meetings, publications in journals, theses or dissertations."

All scientific contributors to the Study have a responsibility to ensure that results of scientific interest arising from Study are appropriately published and disseminated. The Sponsor has a firm commitment to publish the results of the Study in a transparent and unbiased manner without consideration for commercial objectives.

To maximise the impact and scientific validity of the Study, data shall be consolidated over the duration of the study, reviewed internally among all investigators and not be submitted for publication prematurely. Lead in any publications arising from the Study shall lie with the Sponsor in the first instance.

18.1 Before the official completion of the Study,

All publications during this period are subject to permission by the Sponsor. If an investigator wishes to publish a sub-set of data without permission by the Sponsor during this period, the Study Steering Committee shall have the final say.

Exempt from this requirement are student theses that can be submitted for confidential evaluation but are subject to embargo for a period not shorter than the anticipated remaining duration of the study.

18.2 Up to 180 days after the official completion of the study

During this period the Chief Investigator shall liaise with all investigators and strive to consolidate data and results and submit a manuscript for peer-review with a view to publication in a reputable academic journal or similar outlet as the Main Publication.

- The Chief Investigator shall be senior and corresponding author of the Main Publication.
- Insofar as compatible with the policies of the publication outlet and good academic practice, the other Investigators shall be listed in alphabetic order.
- Providers of analytical or technical services shall be acknowledged, but will only be listed as co-authors if their services were provided in a non-routine manner as part of a scientific collaboration.
- Members of the Steering Group shall only be acknowledged as co-authors if they contributed in other capacities as well.
- If there are disagreements about the substance, content, style, conclusions, or author list of the Main Publication, the Chief Investigator shall ask the Steering Group to arbitrate.

18.3 Beyond 180 days after the official completion of the study

After the Main Publication or after 180 days from study end date any Investigator or group of investigators may prepare further publications. In order to ensure that the Sponsor will be able to make comments and suggestions where pertinent, material for public dissemination will be submitted to the Sponsor for review at least sixty (60) days prior to submission for publication, public dissemination, or review by a publication committee. Sponsor's reasonable comments shall be reflected. All publications related to the Study shall credit the Chief and Co-Investigators as co-authors where this would be in accordance with normal academic practice and shall acknowledge the Sponsor and the Funders.

19 Statement of compliance

The study will be conducted in compliance with the protocol, Sponsor's Standard Operating Procedures (SOPs), and the applicable regulatory requirement(s).

The study conduct shall comply with all relevant laws of the EU if directly applicable or of direct effect and all relevant laws and statutes of the UK country in which the study site is located including but not limited to, the Human Rights Act 1998, the Data Protection Act 2018, the Mental Capacity Act 2005, the Human Tissue Act 2004, the Health Service (Control of Patient Information) Regulations 2002 and the UK Policy Framework for Health and Social Care Research.

This study will be conducted in compliance with the protocol approved by the REC. No deviation from the protocol will be implemented without the prior review and approval of the Sponsor and REC except where it may be necessary to eliminate an immediate hazard to a research subject. In such case, the deviation will be reported to the Sponsor and REC as soon as possible.

20 List of protocol appendices

Appendix 1 Summary of Protocol Amendment or Revision History

Appendix 2 Definition of target populations

Appendix 3 Detailed inclusion / exclusion criteria

References

1. Cross Government Group On Gas Safety And Carbon Monoxide (CO) Awareness. Annual Report 2014-2015. http://www.hse.gov.uk/gas/domestic/cross-government-group.htm

2. Reducing the risk of carbon monoxide poisoning over winter [press release]. http://webarchive.nationalarchives.gov.uk/20140714084352/http://www.hpa.org.uk/NewsCentre/NationalPressReleases/2012PressReleases/121119Reducingtheriskofcarbonmonoxidepoisoning/#1. , 19 November 2012 2012

3. Carbon monoxide poisoning sends 4,000 people to A&E each year [press release]. https://www.gov.uk/government/news/carbon-monoxide-poisoning-sends-4-000-people-to-a-e-each-year, 21/11/2011 2011.

4. Heckerling P, Leikin J, Maturen A, Terzian G, Segarra D. Screening hospital populations from the emergency department for occult carbon monoxide. Am J Emerg Med. 1990;8:301-4.

5. Whincup P, Papacosta O, Lennon L, Haines A. Carboxyhaemoglobin levels and their determinants in older British men. BMC Public Health. 2006;6:189.

6. Partridge R, Chee K, Sunar S. Non-invasive carboxyhemoglobin monitoring: screening Emergency Department patients for carbon monoxide exposure. Resp Care Open Forum Abstracts. 2006;51:1332.

7. Hampson N. Emergency Department visits for carbon monoxide poisoning in the Pacific Northwest. J Emerg Med. 1998;16:695-8.

8. Calverley P, Leggett R, Flenley D. Carbon monoxide and exercise tolerance in chronic bronchitis and emphysema. Br Med J. 1981;283:878-80.

9. Dolan M, Haltom T, Barrows G, Short C, Ferriell K. Carboxyhemoglobin levels in patients with flu-like symptoms. Ann Emerg Med. 1987;16:782-6.

10. Balzan M, Cacciottolo J, Mifsud S. Unstable angina and exposure to carbon monoxide. Postgrad Med J. 1994;70:699-702.

11. Balzan M, Agius G, Debono A. Carbon monoxide poisoning: easy to treat but difficult to recognise. Postgrad Med J. 1996;72:470-3.

12. Heckerling P, Leikin J, Maturen A, Perkins J. Predictors of occult carbon monoxide poisoning in patients with headache and dizziness. Ann Intern Med. 1987;107:174-6.

13. Heckerling P. Occult carbon monoxide poisoning: a cause of winter headache. Am J Emerg Med. 1987;5:201-4.

14. Heckerling P, Leikin J, Maturen A. Occult carbon monoxide poisoning: validation of a prediction model. Am J Med. 1988;84:251-6.

15. Heckerling P, Leikin J, Terzian C, Maturen A. Occult carbon monoxide poisoning in patients with neurological illness. Clin Toxicol. 1990;28:29-44.

16. Donaldson L, Mulally S. Carbon monoxide: the forgotten killer. Department of Health; 2002.

17. Volans G. Neuropsychological effects of chronic exposure to carbon monoxide in indoor air. Final report to the Department of Health. London Carbon Monoxide Group; 2006.

18. Kar-Purkayastha I, Finlay S, Murray V. Low-level exposure to carbon monoxide. Br J Gen Prac. 2012;62:404.

19. Clarke S, Keshishian C, Murray V, Kafatos G, Ruggles R, Coultrip E, et al. Screening for carbon monoxide exposure in selected patient groups attending rural and urban emergency departments in England: a prospective observational study. BMJ Open. 2012;2:e000877.

20. Croxford B. Gas appliance check project. Bartlett School of Graduate Studies, University College London 2007.

21. Hao H, Zhou H, Zhang Z, Yu Z. Headspace GC-MS detection of carbon monoxide in decomposed blood and hepatic tissues. J For Sci Criminol. 2013;1(3):1-4.

22. Sowole M, Vuong S, Konermann L. Interactions of hemoglobin and myoglobin with their ligands CN-, CO, and O2 monitored by electrospray ionization-mass spectrometry. Analytical Chemistry. 2015;87:9538-45.

23. Public Health England. Diagnosing poisoning: carbon monoxide. 2015. https://www.gov.uk/government/publications/carbon-monoxide-co-algorithm-to-diagnose-poisoning

24. Mandal S, Ruggles R, Leonardi G, Goodfellow F, Bradley N, Murray V. Developing best practice response to carbon monoxide incidents: a toolkit for health protection frontline staff. Public Health. 2011;125:148-156.

25. Buderer N. Statistical Methodology: I. Incorporating the Prevalence of Disease into the Sample Size Calculation for Sensitivity and Specificity. Academic Emergency Medicine. 1996;3:895-900.

26. van der Flier J, Scheltens P. Epidemiology and risk factors of dementia. Neurol Neurosurg Psychiatry. 2005;76: (Suppl V):v2–v7.

Appendix 1: definition of target populations

Patients presenting to the study sites with the following presenting complaints will be considered for inclusion to both parts of the study:

1. Cardiac chest pain
2. Non-traumatic headache
3. Seizures
4. Flu-like symptoms
5. Syncope/Presyncope

These symptoms were chosen because they are known to be possible features of carbon monoxide exposure. These are defined as follows:

1 Cardiac chest pain:

Cardiac chest pain or Acute Coronary Syndrome (ACS) can be defined based on history and examination, ECG findings and/or elevated blood markers [1]

For the purpose of this study, cardiac chest pain in the ED will be defined as any patient referred to the medical accepting team as suffering from ACS. The researcher will be expected to follow these patients up to ascertain if this was indeed the final diagnosis.

2. Non-traumatic headaches:

Headaches are classified by the ICHD-II as [2]:

- Primary headache disorders
    - Migraine
    - Tension type headache
    - Cluster headache and other trigeminal autonomic cephalagias
    - Primary stabbing, cough, exertional headache
    - Headache associated with sleep
    - Primary thunderclap headache
    - New daily persistent headache
- Secondary headache disorders
    - Head and neck trauma
    - Cranial and cervical vascular disorders
    - Nonvascular intracranial disorder
    - A substance or its withdrawal
    - Psychiatric disorder
    - Other facial or cranial structures
    - Disorders of homeostasis

For the purpose of the study, only primary headache disorders will be included.

3. Seizures:

For the purpose of the study, all patients with seizures regardless of a past medical history of aetiology, will be included.

4. Flu-like illness:

For this study will be based on the following symptoms as defined by NHS Choices [3].

- Sweating and feeling feverish
- General muscle aches and pains
- General tiredness
- Dry, chesty cough
- Sneezing
- Difficulty sleeping

Although CO poisoning is not classically associated with the development of pyrexia, the presence of a raised temperature will not be used as an exclusion criterion: it can be argued that patients with a viral infection may be at higher risk of CO exposure by staying at home and turning on/up the heating. Also, there is a suggestion that CO exposure is associated with an increased frequency of respiratory infections [4]

5. Syncope/Presyncope:

Syncope is defined as the transient, self-limiting loss of consciousness associated with loss of postural tone [5]. It results from global cerebral ischaemia and is associated with rapid, spontaneous recovery. It is usually, but not inevitably preceded by a prodrome where the patient feels that they are about to lose consciousness.

Presyncope is less well-defined but involves a transient alteration of consciousness where the patient feels that they are about to lose consciousness without actually losing it [6]. In many ways it is the prodrome of syncope without progressing to loss of consciousness or postural tone.

References

1. Thygesen K, Alpert JS, Jaffe AS, et al. Third Universal Definition of Myocardial Infarction. Circulation. 2012; 126; 2020-2035.
2. Headache classification subcommittee of the international headache society. The international classification of headache disorders. 2nd edition. Cephalagia 2004; 1:24
3. NHS Choices http://www.nhs.uk/Conditions/Flu/Pages/Symptoms.aspx
4. Estrella B, Estrella R, Oviedo J et al. Acute respiratory diseases and carboxyhemoglobin status in school children of Quito, Ecuador. Environ Health Perspectives
5. Walsh K, et al. Syncope: diagnosis and management. Curr Probl Cardiol. 2015; 40; 51-86
6. Pienado, RP. Is the prognostic significance of presyncope the same as for syncope? Rev Esp Cardiol. 2004; 57; 613-616.

Appendix 2: detailed inclusion / exclusion criteria

Inclusion Criteria	Exclusion Criteria
Cardiac chest pain:<br>• Diagnosed as ACS / STEMI and referred to responsible admitting team	• Other cause for pain identified (e.g. pulmonary embolism, pneumothorax, pneumonia, musculoskeletal)
Non-traumatic headache:<br>• Migraine<br>• Tension type headache<br>• Cluster headache and other trigeminal autonomic cephalagias<br>• Primary stabbing, cough, exertional headache<br>• Headache associated with sleep<br>• Primary thunderclap headache<br>• New daily persistent headache	• Head and neck trauma<br>• Cranial and cervical vascular disorders<br>• Nonvascular intracranial disorder, e.g. tumours<br>• A substance or its withdrawal<br>• Infection<br>• Other facial or cranial structure problems e.g. sinusutis<br>• Disorders of homeostasis
Seizures:<br>• Witnessed tonic-clonic seizures	• Post head injury<br>• Known space occupying lesion<br>• Intracranial haemorrhage
Flu-like illness:<br>(>1 of the following)<br>• Sweating and feeling feverish<br>• Dry, chesty cough<br>• General muscle aches and pains<br>• General tiredness<br>• Sneezing<br>• Difficulty sleeping	• Isolated coryzal symptoms (rhinitis, sneezing, or sore throat)<br>• Clinical features suggestive of specific focus of infection<br>• Otalgia<br>• Pustular tonsillitis<br>• Purulent sputum<br>• Diarrhoea<br>• NB The presence of fever is NOT an exclusion criterion
Syncope<br>• Transient, self-limiting loss of consciousness with loss of postural tone<br>• Presyncope<br>• Transient alteration of consciousness where the patient feels that they are about to lose consciousness without actually losing it<br>• Symptoms may include:<br>o Dizziness<br>o Lightheadedness<br>o Weakness<br>o Blurred vision<br>o Tunnel vision<br>o Diaphoresis<br>o Nausea	• Trauma<br>• Previously diagnosed situational syncope
