# Supplementary material for: Screening patients for unintentional carbon monoxide exposure in the Emergency Department: a cross-sectional multi-centre study
Source: J Public Health (Oxf). 2023 Jan 31;45(3):553–9. doi: 10.1093/pubmed/fdad007 (PMC10470336; doi:10.1093/pubmed/fdad007)
Supplement: Supplementary_material_2_full_inclusion_fdad007 [file supplementary_material_2_full_inclusion_fdad007.pdf]

Supplementary material 1: detailed inclusion / exclusion criteria

Inclusion Criteria	Exclusion Criteria
Cardiac chest pain:<br>• Diagnosed as ACS / STEMI and referred to responsible admitting team<br>• Investigation for cardiac chest pain	• Other cause for pain identified (e.g. pulmonary embolism, pneumothorax, pneumonia, musculoskeletal)
Non-traumatic headache:<br>• Migraine<br>• Tension type headache<br>• Cluster headache and other trigeminal autonomic cephalagias<br>• Primary stabbing, cough, exertional headache<br>• Headache associated with sleep<br>• Primary thunderclap headache<br>• New daily persistent headache	• Head and neck trauma<br>• Cranial and cervical vascular disorders<br>• Nonvascular intracranial disorder, e.g. tumours<br>• A substance or its withdrawal<br>• Infection<br>• Other facial or cranial structure problems e.g. sinusutis<br>• Disorders of homeostasis
Seizures:<br>• Witnessed tonic-clonic seizures	• Post head injury<br>• Known space occupying lesion<br>• Intracranial haemorrhage
Flu-like illness:<br>(>1 of the following)<br>• Sweating and feeling feverish<br>• Dry, chesty cough<br>• General muscle aches and pains<br>• General tiredness<br>• Sneezing<br>• Difficulty sleeping	• Isolated coryzal symptoms (rhinitis, sneezing, or sore throat)<br>• Clinical features suggestive of specific focus of infection<br>• Otalgia<br>• Pustular tonsillitis<br>• Purulent sputum<br>• Diarrhoea<br>• NB The presence of fever is NOT an exclusion criterion
Syncope<br>• Transient, self-limiting loss of consciousness with loss of postural tone<br>• Presyncope<br>• Transient alteration of consciousness where the patient feels that they are about to lose consciousness without actually losing it<br>• Symptoms may include:<br>○ Dizziness<br>○ Lightheadedness<br>○ Weakness<br>○ Blurred vision<br>○ Tunnel vision<br>○ Diaphoresis<br>○ Nausea	• Trauma<br>• Previously diagnosed situational syncope
